# The Singular Epidemiology of Plasmacytoma and Multiple Myeloma in French Guiana

**DOI:** 10.3390/cancers16010178

**Published:** 2023-12-29

**Authors:** Laure Manuella Imounga, Kinan Drak Alsibai, Juliette Plenet, Qiannan Wang, Beatrice Virjophe-Cenciu, Pierre Couppie, Nadia Sabbah, Antoine Adenis, Mathieu Nacher

**Affiliations:** 1 Registre des Cancers de Guyane (RCan Guyane), Département Research, Innovation et Santé Publique (DRISP), Cayenne Hospital Center Andrée Rosemon, 97300 Cayenne, French Guiana; laure.imounga@ch-cayenne.fr (L.M.I.); qiannan.wang@ch-cayenne.fr (Q.W.); antoine.adenis@ch-cayenne.fr (A.A.); 2 Département Formation Recherche (DFR) en Santé, Université de Guyane, 97300 Cayenne, French Guiana; pierre.couppie@ch-cayenne.fr; 3 Service d’Anatomie et de Cytologie Pathologiques, Cayenne Hospital Center Andrée Rosemon, 97300 Cayenne, French Guiana; 4 Centre d’Investigation Clinique (CIC, INSERM 1424), Cayenne Hospital Center Andrée Rosemon, 97300 Cayenne, French Guiana; 5 Union Régionale des Professionnels de Santé (URPS), 97300 Cayenne, French Guiana; juliette.plenet@orange.fr; 6 Hôpital de Jour Adultes, Cayenne Hospital Center Andrée Rosemon, 97300 Cayenne, French Guiana; beatrice.virjoghe@ch-cayenne.fr; 7 Service de Dermatologie, Cayenne Hospital Center AndréeRosemon, 97300 Cayenne, French Guiana; 8 Service d’Endocrinologie et Diabétologie, Cayenne Hospital Center Andrée Rosemon, 97300 Cayenne, French Guiana; nadia.sabbah@ch-cayenne.fr

**Keywords:** multiple myeloma, plasmacytoma, French Guiana, incidence, mortality, Latin America

## Abstract

**Simple Summary:**

The objective of the present study was to compare the incidence of and mortality due to plasmacytoma and multiple myeloma in French Guiana with mainland France and with Latin American countries, and to determine whether the epidemiological profile in our territory was South American or European. The incidence of these tumors was higher in French Guiana than in other countries in Latin America. The incidence has increased in the past decade, especially among women. The underlying explanation may be a large population with African ancestry and a high prevalence of obesity in our territory, particularly among women.

**Abstract:**

Background: The objective was to review a decade of plasmacytoma (PC) and multiple myeloma (MM) data from French Guiana, and to study its spatial and temporal trends. Methods: This was a retrospective study of MM and PC between January 2005 and December 2014 using cancer registry data, including age-standardized incidence and mortality rates. Results: There were 110 cases of PC and MM (62 women and 48 men), representing the eighth most frequent malignancy in French Guiana. PC and MM were much more common in females. In men, 79% of cases occurred at ≥55 years, and in women, 90% of cases occurred at ≥50 years. The median age at diagnosis was 60 years for men and 66 years for women, while it was 72 years for men and 75 years for women in mainland France. The incidence rate standardized to the world population was 5.9 patients of PC and MM per 100,000 men/year and 7.8 per 100,000 women/year. Conclusions: In our territory, the incidence of PC and MM was higher and patients were diagnosed at a substantially younger age than in mainland France. Women had a greater incidence than men, and there was an increasing temporal trend of incidence among women. African ancestry and the frequency of obesity, notably among women, could have contributed to this observation.

## 1. Introduction

Multiple myeloma (MM) is a clonal proliferative disorder of plasma cells, and is the second most frequent hematologic malignancy after non-Hodgkin’s lymphoma. More rarely, plasma cell proliferation may appear as a solitary lesion called plasmacytoma (PC) and can be found in bone (solitary PC) or in soft tissues (extramedullary PC) [1,2].

The known MM and PC risk factors are family history, male sex, exposure to radiation or chemicals, monoclonal gammopathy of undetermined significance (MGUS), an age greater than 60 years, and African ancestry [3]. More recently, excess weight and obesity have also been identified as potential risk factors for MM, as excess body weight during midlife has been associated with a risk of progression of MGUS to MM [1,4,5].

With greater access to health services and diagnosis, and better case reporting, incidence of MM and PC has gradually increased. Despite the lower health expenditure in Latin America and the often younger demographic structure, incidence and mortality rates in Latin America are greater than those in Europe, with heterogeneity between countries [6,7,8]. French Guiana is a French overseas territory situated between Brazil and Suriname. It has a young population (median age 22 years) and is ethnically mixed, with a majority of persons of African ancestry. Despite having the highest gross domestic product (GDP) per capita in Latin America, a large proportion of the population is socially precarious. As a French territory, it benefits from high health expenditures for the population, and, despite low health professional density, it has connections with large cancer centers in France where patients are often referred for treatment. For patients treated in French Guiana, all approved drugs used in French cancer treatment protocols are available. French Guiana also has a regional cancer registry that allows producing reliable indicators for cancers, an important asset to improve knowledge of the distinct local epidemiology.

The objective of the present study was to compare the incidence of and mortality due to plasmacytoma (PC) and multiple myeloma (MM) with mainland France and with Latin America, and to determine whether the epidemiological profile in French Guiana was South American or European. 

## 2. Materials and Methods

Since its creation in 2005, the main mission of the Cancer Registry of French Guiana has been to carry out a regular, and as exhaustive as possible, inventory of the epidemiological situation with regard to cancer in the whole territory [9,10,11]. It relies on data from hundreds of notification and information sources, the vast majority of which are outside French Guiana. Within French Guiana, collection mainly focuses on the three public hospitals in Cayenne, Kourou, and Saint Laurent du Maroni. The registration and coding of tumors by research technicians comply with the rules established by the networks of French and European registers, FRANCIM and ENCR, respectively [12,13].

The registry has continuously and exhaustively listed new cases of cancer diagnosed since 1 January 2003, corresponding to invasive and/or in situ tumors of patients residing in French Guiana, regardless of the cancer location [9,10,11].

The registry is based on National Information Systems Medical Program (PMSI) data, transmitted by FRANCIM, and the technical agency for information on hospitalization (ATIH) [11]. These files mention all hospital stays of anonymized patients, with CIM10 codes and a corresponding location code in French Guiana, which allows the registry to query French health structures within and outside of French Guiana [10,11]. Data collection also relies on local physicians, biologists, pathologists, medical information departments (DIMs) and hospital archives for access to medical records of patients with cancer.

The registry database is sent annually to the Hospices Civils de Lyon (HCL) and to the International Agency for Research on Cancer (IARC) [11].

The cancer registry of French Guiana has received approval from the National authorities (Commission Nationale Informatique et Libertés).

For the present analysis, we searched for ICD codes C90 and C88 between 1 January 2005 and 31 December 2014. Data were analyzed using Stata (Stata Corporation, College Station, TX, USA). Direct standardization of age structure was performed using the world population. 

## 3. Results

**Figure 1:**
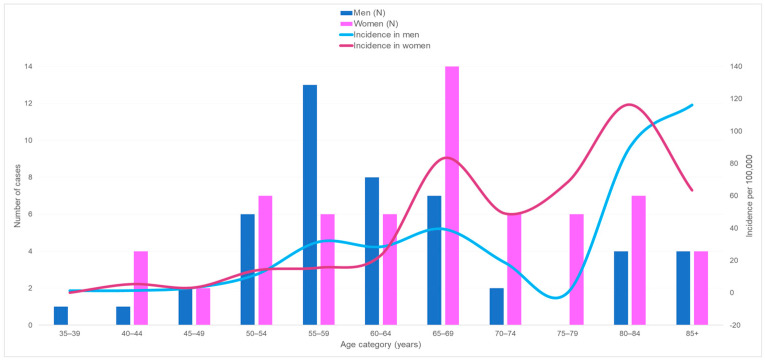
Number of new cases of multiple myeloma and plasmacytoma and crude incidence rates by age and sex, between 2005 and 2014 in French Guiana.

Between 2005 and 2014, there were 110 patients with PC and MM (62 women and 48 men (Figure 1)), representing the eighth most frequent malignancy in French Guiana. PC and MM were more common in the female population. In men, 79% of cases occurred at age 55 and over, and in women, 90% of cases occurred at 50 years and over. The median age at diagnosis was 60 years for men and 66 years for women, while in mainland France, in 2012, it was 72 years for men and 75 years for women. Of 107 patients with PC or MM, 43 (40.2%) were foreign born (outside French Guiana and mainland France). Data about the place of birth were missed for three patients.

### 3.1. Incidence

Figure 1 shows that between 2005 and 2014, the specific crude incidence was very low in both sexes before age 50 (less than six cases per 100,000) and then increased with a peak at 65–69 years (39.5 for men and 83.2 for women). After declining from age 70 in both populations, the incidence started to rise again, reaching 116.4 and 90 in women and men aged 80 to 84 years, respectively. After 84 years, it continued to grow in the male population, while it decreased in the female population. 

The incidence rate standardized to the world population over the 2005–2014 period was 5.9 cases of PC and MM per 100,000 man-years and 7.8 per 100,000 woman-years, whereas it was 4.2 and 2.9 in France in 2012, respectively. Regarding trends, between 2005–2009 and 2010–2014 the average incidence slightly increased in men from 5.18 to 6.7 cases per 100,000, and increased in women from 5.88 to 9.5 cases per 100,000.

**Figure 2:**
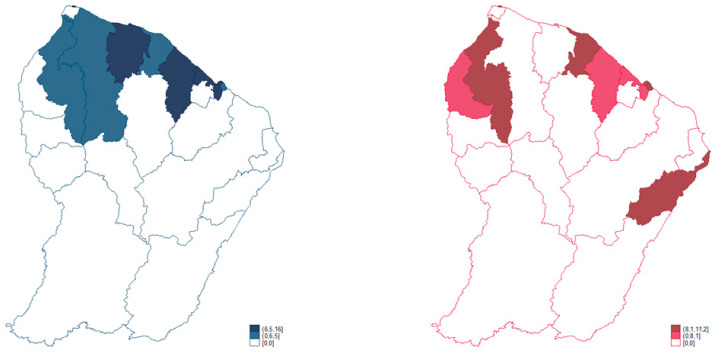
Spatial distribution of standardized rates of multiple myeloma and plasmacytoma for men (**left**) and women (**right**) between 2005 and 2014 in French Guiana. French Guiana Cancer Registry. 2005–2014. Standardized incidence rate per 100,000 man-years; reference population: World.

At the regional level during this decade, among men (Figure 2), the standardized incidence rates were zero for more than half of the municipalities (Apatou, Awala-Yalimapo, Camopi, Grand-Santi, Maripasoula, Montsinéry-Tonnegrande, Ouanary, Papaïchton, Régina, Roura, Saint-Elie, Saint-Georges, and Saül), and the maximum was observed for Macouria-Tonate (Center coastal) at 16.0 per 100,000 man-years.

In addition, six other municipalities had higher incidence rates than the regional average for the male population: Iracoubo, Kourou, and Sinnamary (Savanas) had respective rates of 10.5, 6.9, and 6.5; and Cayenne, Matoury, and Rémire-Montjoly (Center coastal) had respective rates of 8, 6.6, and 6.4.

In women (Figure 2), at the regional level during this decade, the standardized incidence rates were zero for more than half of the municipalities of French Guiana (Awala-Yalimapo, Camopi, Grand-Santi, Iracoubo, Mana, Maripasoula, Montsinéry-Tonnegrande, Ouanary, Papaïchton, Régina, Roura, and Saint-Elie), and the maximum rate was observed for Saint-Laurent-du-Maroni (West) at 11.2 per 100,000 woman-years.

Five other municipalities had higher incidence rates than the regional average for the female population: Sinnamary’s (Savanas) rate was 9.6; Cayenne, Rémire-Montjoly, and Matoury (Center coastal) had respective rates of 9.4, 8.4, and 8.1; and Saint-Georges’ (East) rate was 9.2.

### 3.2. Mortality

**Figure 3:**
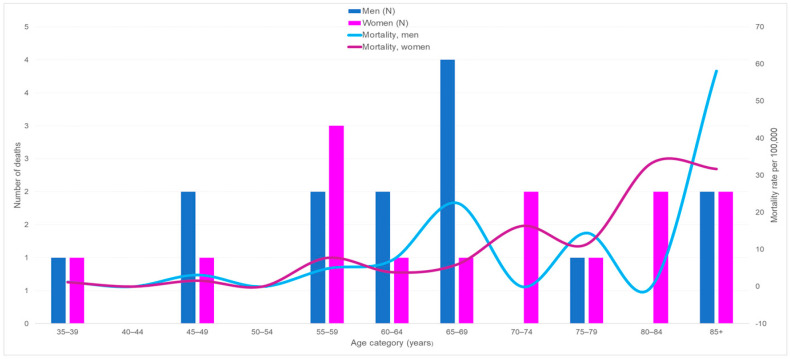
Number of deaths from plasmacytoma and multiple myeloma and mortality rates by age and sex in French Guiana between 2005 and 2014.

Figure 3 shows the number of deaths from PC and MM by age group and mortality rates per 100,000. Throughout the decade, mortality seemed to increase among women. Among men, it declined from 2.2 per 100,000 in 2005–2009 to 1.5 per 100,000 in 2010–2014; among women it increased from 1.04 per 100,000 in 2005–2009 to 1.9 per 100,000 in 2010–2014. Of 23 deaths with available information, nine deceased persons (39.1%) were foreign born.

### 3.3. International Comparisons

**Figure 4:**
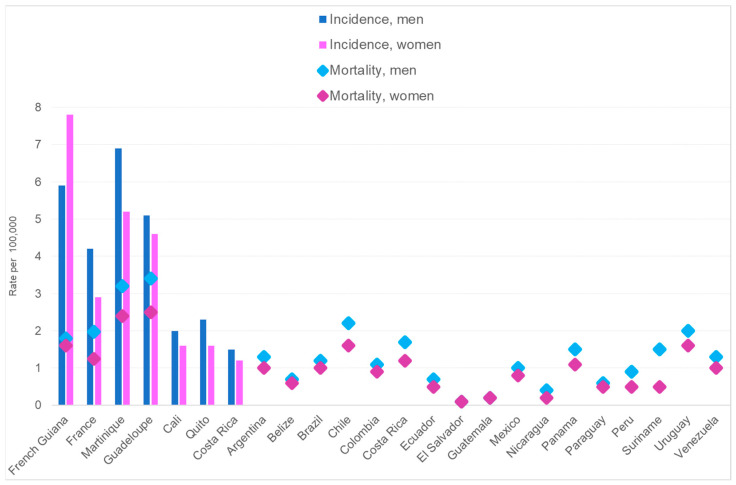
Standardized incidences of and mortalities from multiple myeloma.

Figure 4 shows international comparisons for incidence and mortality. The highest incidence was in French Guiana, and the inversion of the usual higher incidence and mortality in men was a notable feature. Despite this high incidence, mortality was similar to that of France and lower than in Martinique and Guadeloupe.

## 4. Discussion

The hypothesis we aimed to test was that plasmacytoma and multiple myeloma in French Guiana were more South American than French. However, what we observed was neither a French pattern nor a South American one. The present results show that the incidence of PC and MM was significantly higher in French Guiana than in mainland France or in other Latin American countries. Moreover, striking facts were the female preponderance and its recent increase, observations that are contrary to the usual predominance of MM in males [2,3,14,15,16]. The median age was substantially younger in French Guiana than in France [14]. Incidence increased with age, and throughout the decade of observation the overall trend was an increase in the incidence of PC and MM among women. The spatial repartition within French Guiana was heterogeneous, with areas such as Western French Guiana, Eastern French Guiana, and the middle coastal area having greater incidences among women. Unfortunately, the cancer registry does not provide data regarding clinical and laboratory analyses. Therefore, we were not able to classify myeloma into smoldering or active types.

In French Guiana, nearly 65–70% of the population is of African ancestry [6], which increases the risk of MM. The ethnic distribution of the population in the territory is not homogenous, which may explain the regional heterogeneities observed in French Guiana. French legislation makes it difficult to record ethnicity in medical research; therefore, we could not know the ancestral origins of patients with certainty, which was a limitation. Other countries in Latin America do have large populations of African ancestry, but in lower proportions than French Guiana does [17,18,19,20]. Guadeloupe and Martinique, the two French overseas territories nearest to French Guiana, which have an even greater proportion of their populations of African descent [21,22,23], have lower standardized incidence rates than French Guiana does (in males, 5.12, 6.94, and 7.03, respectively, and in females, 5.1, 6.9, and 7, respectively) [24]. In contrast, age-standardized mortality rates seemed to be greater in males in Guadeloupe or Martinique than in French Guiana, (3.4. 3.2, and 1.8, respectively); in females the same pattern was observed (2.5, 2.4, and 1.6, respectively) [17]. 

There have been observations of spatial heterogeneity of MM within Martinique that led to a hypothesis that organochlorine pesticide exposure was the explanation for the observed pattern [25]. French Guiana does not have such an organochlorine problem; therefore, the explanation for the greater incidence, notably among women, and lower mortality remains elusive. Some authors have suggested additional risk factors, including obesity, low fish consumption, low green vegetable consumption, acquired immune deficiency syndrome (AIDS), and herpes zoster infection [26]. 

Obesity (body mass index > 30 kg/m^2^) is a growing problem that affects 23% of women and 13% of men in French Guiana, a difference that may partly explain the observed differences between men and women, and the temporal trends [27,28]. However, we can only speculate that this is a major explanation because cancer registry data do not provide this information. The relative increase in standardized incidence in Western French Guiana and Eastern French Guiana raised the possibility that immigrants, notably women, would declare residency in one of the border towns, hence benefitting from care and increasing incidence rates. However, this seems unlikely because it has not been observed for other types of cancers. Although territorial differences in access to care could in theory partly explain spatial differences in incidence, it seems unlikely given the seriousness of the disease and the broad network of free health centers with the ability to conduct blood analyses and make referrals to Cayenne hospital. The present results point towards future areas of more in-depth research to better understand the observed singularities of PC and MM.

## 5. Conclusions

The incidence of PC and MM in French Guiana was high and patients were diagnosed at a substantially younger age than they were in mainland France. For reasons that are not clear, the analysis of the 2005–2014 decade showed a singular pattern, with a greater incidence among women than among men, and an increasing temporal trend of incidence among women. The frequent African ancestry and increasing frequency of obesity, notably among women in our territory, could have contributed to this observation. 

## Data Availability

The authors confirm that data supporting the findings of this study are available within the article.
